# Enhanced Endogenous GABA Biosynthesis Modifies Fruit Production and GABA Accumulation in a Medium‐Sized Tomato Cultivar Under Salt Stress

**DOI:** 10.1002/pei3.70187

**Published:** 2026-07-15

**Authors:** Toon Suzuki, Mariko Takayama, Hiroshi Ezura

**Affiliations:** ^1^ Degree Program in Agro‐Bioresources Science and Technology, Graduate School of Science and Technology University of Tsukuba Tsukuba Ibaraki Japan; ^2^ Institute of Life and Environmental Sciences University of Tsukuba Tsukuba Ibaraki Japan; ^3^ Tsukuba Plant Innovation Research Center (T‐PIRC) University of Tsukuba Tsukuba Ibaraki Japan; ^4^ Sanatech Life Science Co Ltd Minato‐ku Tokyo Japan

**Keywords:** abiotic stress, CRISPR‐Cas9, dry matter, genome editing, metabolic engineering, salt stress, *Solanum lycopersicum*, stress tolerance

## Abstract

While exogenous γ‐aminobutyric acid (GABA) enhances plant stress tolerance, fruit responses to increased endogenous GABA biosynthesis remain unclear. Although high‐GABA traits mitigate productivity loss in cherry tomatoes under salt stress, salinity responses differ among cultivars, and whether this occurs in medium‐sized tomatoes remains unknown. We tested whether endogenous GABA enrichment maintains production and promotes dry matter and GABA accumulation under salt stress. The medium‐sized tomato ‘Esprosso’ (ER), and its genome‐edited high‐GABA line (ERHG) were grown under control and salt stress conditions. Under salt stress, fruit fresh weight decreased by 43.4% in ER and 31.5% in ERHG, whereas fruit number per plant increased by 33.3% in ER and 71.1% in ERHG. Due to this compensatory increase, mean yield changes (+2.6% in ER and +11.1% in ERHG) were not statistically significant. Dry matter content increased by 0.6 percentage points (pp) in ER and 1.5 pp. in ERHG, showing a significant genotype × salt stress interaction. Salt stress elevated GABA concentration by 64.5% in ER and 74.4% in ERHG (from 141.5 to 246.8 mg 100 g^−1^ FW), increasing per‐fruit GABA content by 22.8% in ERHG. Total soluble solids (TSS) and ascorbic acid increased under salt stress in both genotypes, indicating the high‐GABA trait did not reduce TSS, whereas proline showed a genotype‐dependent response. These results suggest that enhanced endogenous GABA biosynthesis modifies fruit responses to salt stress by promoting GABA and dry matter accumulation and altering osmolyte‐related metabolism without reducing TSS, potentially contributing to high‐value tomato production under controlled salinity.

## 1 Introduction

Soil salinization, caused by climate change and intensive agriculture, threatens global food security (Yang and Guo 2018; Corwin 2021). Salt stress fundamentally impairs plant growth through primary osmotic and ionic stresses (Munns and Tester 2008). An increase in the Na^+^ concentration in the soil reduces the osmotic potential in the rhizosphere, restricting water uptake, which impairs photosynthesis and inhibits cell expansion (Hasanuzzaman et al. 2021; Van Zelm et al. 2020; Zhao et al. 2021). To counter this, plants synthesize osmolytes, such as amino acids and soluble sugars, to maintain cell turgor pressure (Flowers et al. 2015; Sanchez et al. 2008). Ionic toxicity from excessive Na^+^ uptake can denature proteins, inhibit enzyme activity, and antagonize the essential nutrient potassium (K). When K^+^ uptake is inhibited, the intracellular ionic balance is disrupted (Assaha et al. 2017; Kronzucker et al. 2013; Pardo et al. 2006). These primary stresses induce the overproduction of reactive oxygen species (ROS), causing oxidative damage to cell membranes and nucleic acids (Zushi and Matsuzoe 2009; Decros et al. 2019). In tomato production, while these complex processes limit vegetative growth and fruit yield (Saito, Matsukura, et al. 2008; Saito, Fukuda, et al. 2008; Zhang et al. 2016), moderate salinity can conversely improve fruit quality traits, including total soluble solids (TSS) and antioxidant compounds (Yin et al. 2010; Massaretto et al. 2018; Roșca et al. 2023). Thus, controlled salinity can serve as a cultivation condition that can modify fruit metabolism and quality.

Plants utilize various defense mechanisms against salinity, among which γ‐aminobutyric acid (GABA) plays a multifaceted role in stress tolerance by acting as an osmolyte to maintain cell turgor, buffering cytoplasmic pH by consuming protons (H^+^) during GABA synthesis, and regulating ROS detoxification (Li et al. 2021; Ansari et al. 2021; Seifikalhor et al. 2019; Yuan et al. 2023). In tomato fruits, GABA is primarily synthesized from glutamate via cytosolic glutamate decarboxylase (GAD) (Takayama and Ezura 2015). Under stress, the metabolic pathway known as the GABA shunt bypasses tricarboxylic acid (TCA) cycle steps by converting GABA to succinic semialdehyde (SSA) via GABA transaminase (GABA‐T) and subsequently to succinate, directly sustaining carbon‐nitrogen balance and respiratory metabolism (Fait et al. 2008; Yuan et al. 2023; Kim et al. 2025). Furthermore, salt stress upregulates the synthesis pathway of proline and polyamines (De la Torre‐González et al. 2018; Hasanuzzaman et al. 2021). Additionally, exogenous GABA priming and genetic overexpression of *SlGAD1* have been shown to enhance tomato salinity tolerance by regulating endogenous amino acids and ROS pathways (Wu et al. 2020; Wang et al. 2024).

In tomato fruit, *SlGAD2* and *SlGAD3* have been identified as the major isozymes catalyzing this reaction (Takayama et al. 2015). Plant GAD contains an autoinhibitory domain at the C‐terminus, which inhibits its enzymatic activity under normal conditions but is relieved under stress (Snedden et al. 1995; Gut et al. 2009). Truncating this domain of *SlGAD3* via genetic modification or genome editing increases both GAD activity and GABA content in fruits (Takayama et al. 2017; Nonaka et al. 2017). Although this trait has been reported to be dominant (Lee et al. 2018) and valuable for biofortification, recent studies note that high GABA mutations can induce growth delays and reduced yield under normal conditions, highlighting potential productivity trade‐offs (Choi et al. 2026). Conversely, introducing this autoinhibitory domain truncation into a cherry tomato cultivar mitigated productivity loss and increased GABA content under salt stress conditions (Suzuki et al. 2025). However, salinity responses differ among tomato cultivars, and whether this protective effect occurs in medium‐sized tomatoes remains unknown, identifying a critical research gap.

Therefore, this study utilized a medium‐sized tomato cultivar to investigate whether truncating the SlGAD3 autoinhibitory domain could enhance salt stress tolerance, focusing on agricultural productivity and dry matter content to evaluate previously observed metabolic trade‐offs. We hypothesized that enhanced endogenous GABA biosynthesis would help maintain fruit production under salt stress, promote further GABA accumulation, and alter fruit metabolic responses, including dry matter accumulation and antioxidant‐ or osmolyte‐related metabolism. To test these hypotheses, we compared the original F_1_ medium‐sized tomato cultivar ‘Esprosso’ (ER) and its genome‐edited high‐GABA line ‘Esprosso High GABA’ (ERHG) under control and salt stress conditions, evaluating fruit production traits, fresh weight, dry matter content, GABA accumulation, TSS, ascorbic acid, and proline content.

## 2 Methods and Materials

### 2.1 Plant Materials, Experimental Design, and Growth Conditions

The original F_1_ medium‐sized tomato ‘Esprosso’ (ER; Sanatech Seed Co. Ltd., Tokyo, Japan) and its genome‐edited line ‘Esprosso High GABA’ (ERHG; Sanatech Life Sciences Co. Ltd., Tokyo, Japan) were cultivated from February to September 2024 using a 2 × 2 factorial design (*n* = 12 biological replicates per treatment, 48 plants in total) in a greenhouse at the University of Tsukuba, Japan.

Seedlings grown under 16‐h light (60 μmol m^−2^ s^−1^)/8‐h dark cycle at 25°C for 6 weeks were transplanted into coconut coir slabs (Coco Bags, Toyotane Co. Ltd., Aichi, Japan) at 15 cm intervals. Plants were irrigated with a commercial nutrient solution (Tank Mix, OAT Agrio Co. Ltd., Osaka, Japan) at 1.5 L plant^−1^ day^−1^. The control group received the solution at an electrical conductivity (EC) of 1.2–1.5 dS m^−1^ from flowering to fruit set, and 1.8–2.4 dS m^−1^ during ripening.

For the salt stress treatment, NaCl was supplemented weekly to the identical nutrient solution, with the EC monitored using an EC meter (Hanna Instruments Japan LLC, Chiba, Japan). To ensure stable plant establishment, a two‐step management approach was implemented: NaCl was introduced after the second inflorescence set to maintain the EC at 6.0 dS m^−1^, and then elevated to at 8.0 dS m^−1^ after fruit enlargement, following practical high‐sugar tomato cultivation in Japan (Saito, Matsukura, et al. 2008; Saito, Fukuda, et al. 2008; Schnitzler and Krauss 2010; Suzuki et al. 2025). Substrate EC was monitored every 14 days; if it exceeded 12.0 dS m^−1^, NaCl supplementation was suspended until the substrate EC fell below dS m^−1^ to prevent extreme ion toxicity (Hanna Instruments Co. Ltd., Chiba, Japan).

### 2.2 Fruit Sampling and GABA Quantification

To assess fruit quality, red‐ripe fruits were harvested from the fourth inflorescence 14 days after reaching the breaker stage to minimize variations in GABA content due to differences in fruit maturity (Akihiro et al. 2008). Fruit samples were collected from randomly selected plants, with five biological replicates (*n* = 5), excluding perimeter plants to eliminate potential edge effects. GABA content was measured using a commercial GABA assay kit (Enzyme Sensor Co. Ltd., Ibaraki, Japan) according to the method described previously by Suzuki et al. (2025). Briefly, frozen fruit samples were homogenized, and the enzymatic reaction was performed following the manufacturer's instructions. The absorbance was measured at 555 nm using a microplate reader (BioTek Instruments, Vermont, USA). The GABA content was determined using a standard calibration curve and was expressed as mg g^−1^ FW or mg fruit^−1^.

### 2.3 Yield, Fruit Number, Fresh Weight and Dry Matter Content

Total yield and fruit number were recorded weekly from June 2 to August 26, 2024, using five plants per treatment as biological replicates (*n* = 5). Cumulative yield was calculated from red fruits weighing more than 10 g and expressed as g FW plant^−1^. For quality traits, five randomly selected red‐ripe fruits per treatment were partitioned: one half for dry weight (DW) determination, one quarter for total soluble solids (TSS), and the remainder frozen in liquid nitrogen and stored at −80°C for GABA measurement. TSS was measured from extracted juice using a digital refractometer (ATAGO Co. Ltd., Tokyo, Japan) and expressed as %. To determine dry matter content (%), the designated fruit halves were oven‐dried at 80°C for 72 h to a constant weight using an electronic balance (Mettler Toledo, Ohio, USA) and calculated as the percentage of DW relative to FW.

### 2.4 Quantification of Ascorbic Acid and Proline Concentrations

Ascorbic acid and proline concentrations were determined using red‐ripe fruits from three biologically independent plants (*n* = 3) per treatment. Fruit samples were immediately frozen in liquid nitrogen and stored at −80°C until analysis.

Ascorbic acid was extracted with 6% metaphosphoric acid and quantified using an IDK Vitamin C assay kit (Immundiagnostik AG, Bensheim, Germany) according to the small‐scale method described by Stevens et al. (2006). The absorbance was measured at 520 nm using a microplate reader (Molecular Devices, San Jose, CA, USA), and the final concentration was calculated from a standard calibration curve and expressed as mg g^−1^ FW.

Proline concentration was determined based on the protocol by Bates et al. (1973) with minor modifications. Briefly, frozen fruit tissue (1 g FW) was homogenized in 3% sulfosalicylic acid, reacted with acid ninhydrin reagent, and extracted with toluene. The absorbance of the toluene phase was measured at 520 nm using a spectrophotometer (Beckman Coulter Inc., CA, USA). The proline concentration was calculated from an L‐proline standard curve and expressed as mg g^−1^ FW.

### 2.5 Statistical Analysis

All statistical analyses were performed via Two‐way analysis of variance (ANOVA). Prior to ANOVA, data normality and homogeneity of variance were verified using the Jarque–Bera and Levene's tests, respectively. Partial eta‐squared (*η*_p_^2^) and Cohen's *d* were calculated to evaluate effect sizes. When a significant interaction was detected, Tukey's honestly significant difference (HSD) test was applied for post hoc multiple comparison (Stahle and Wold 1989). Statistical significance was evaluated using actual *p*‐values, with *p* < 0.05 considered significant. All analyses were conducted using the Real Statistics Resource Pack add‐in for Microsoft Excel (Microsoft Corp., Washington, USA).

## 3 Results

### 3.1 Effects of Salinity on Fruit Yield and Number

**FIGURE 1:**
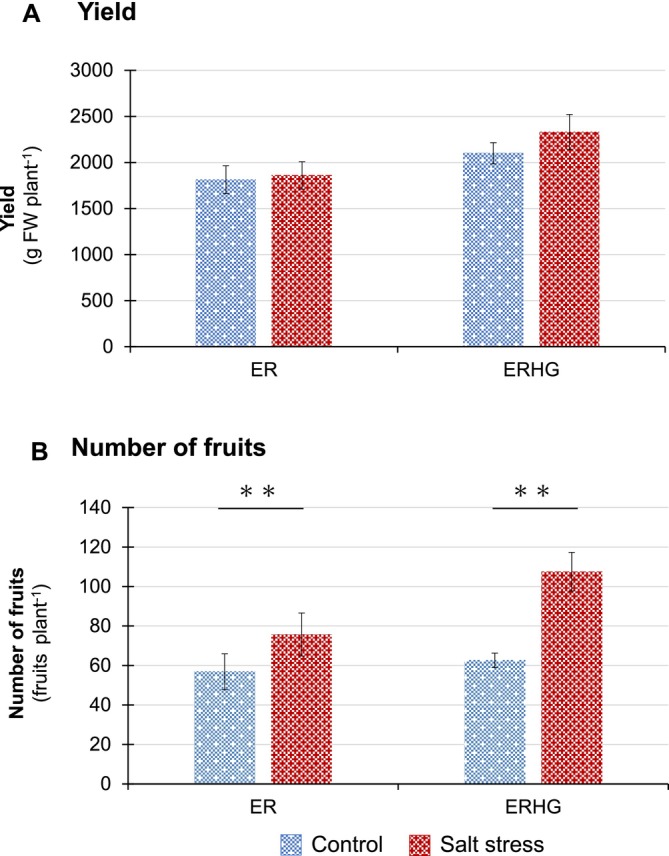
Yield and fruit number of ER and ERHG under salt stress. (A) Yield (total weight of fruit per plant). (B) Number of fruits. All data were determined when fruit reached to the red stage. Results are the mean ± SE (*n* = 5). Asterisks indicate significant differences between control and salt stress conditions, and between ER and ERHG, as determined by a two‐way repeated measures ANOVA (*p < 0.05, **p < 0.01). ER, ‘Esprosso’; ERHG, ‘Esprosso High GABA’; FW, fresh weight; SE, standard error.

Under salt stress, the average fruit yield per plant increased by 2.6% in the ER cultivar and 11.1% in the ERHG cultivar (Figure 1A). Two‐way ANOVA showed no statistically significant differences for the main effects of cultivar, stress, or their interaction (Table S1). However, the stress effect size was larger in ERHG than in ER, suggesting that salt stress may partially elicit the yield potential inherent in the ERHG cultivar.

Concurrently, salt stress increased fruit number by 33.3% in ER and 71.7% in ERHG (Figure 1B). Two‐way ANOVA revealed a significant main effect of stress, whereas the effects of cultivar and their interaction were not significant (Table S1). These results suggest that the maintenance of fruit yield under salt stress was associated with increased fruit number rather than preservation of individual fruit size.

### 3.2 Fruit Fresh Weight and Dry Matter Content

**FIGURE 2:**
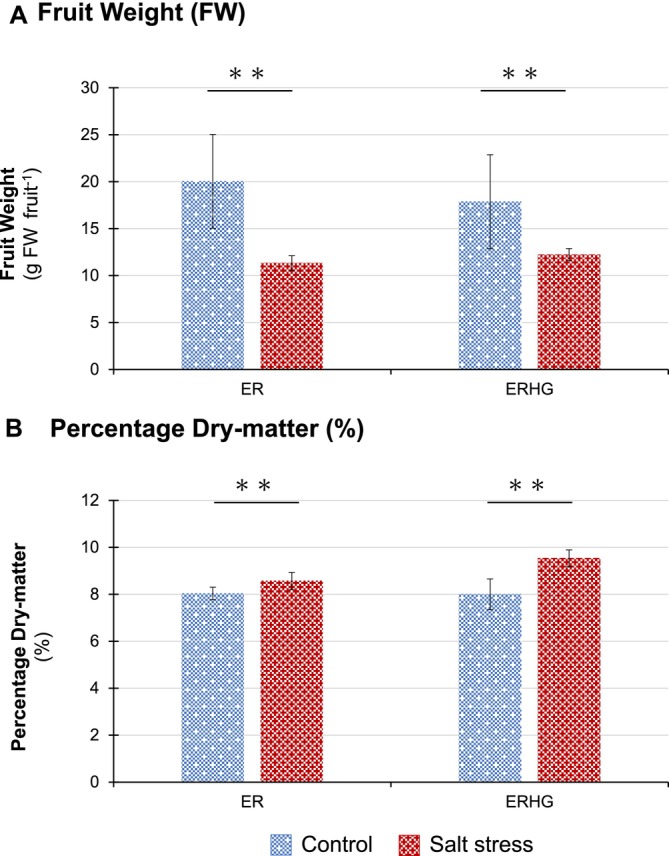
Fruit fresh weight and dry matter percentage of ER and ERHG under salt stress. (A) Fresh weight (gFW per one fruit). (B) Percentage Dry‐matter (%) Red mature fruits were harvested from ER or ERHG plants grown under control or salt stress conditions. Results are the mean ± SE (*n* = 5). Asterisks indicate significant differences between Control and Salt stress conditions, and between ER and ERHG, as determined by a two‐way repeated measures ANOVA (*p < 0.05, **p < 0.01). ER, ‘Esprosso’; ERHG, ‘Esprosso High GABA’; FW, fresh weight; SE, standard error.

Under salt stress, fruit fresh weight decreased by 43.4% in ER and 31.5% in ERHG (Figure 2A). Two‐way ANOVA showed that the main effect of stress on fruit fresh weight was highly significant, whereas the effects of cultivar and their interaction were not significant (Table S1). Nevertheless, the descriptive trend suggests a potential mitigation of salinity‐induced fruit weight loss in ERHG.

In contrast, salt stress elevated fruit dry matter content by 0.6 percentage points in ER and 1.5 percentage points in ERHG (Figure 2B). Two‐way ANOVA showed that both the main effect of stress and the interaction between cultivar and stress were statistically significant, while the main effect of cultivar was not significant (Table S1). These results indicate that ERHG showed a stronger dry matter accumulation response under salt stress.

### 3.3 GABA Accumulation and Concentrating Effects

**FIGURE 3:**
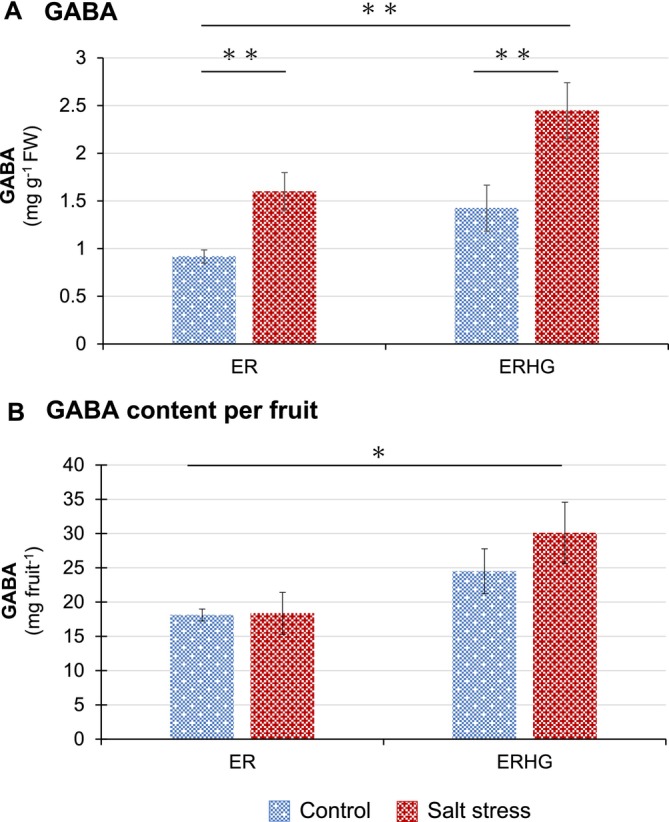
ERHG has the potential to accumulate more GABA in fruits when grown under salt stress condition. (A) GABA content (mg per gFW). (B) GABA content (mg per one fruit). Red mature fruits were harvested from ER or ERHG plants grown under control or salt stress conditions and used for the GABA measurement. Results are the mean ± SE (*n* = 5). Asterisks indicate significant differences between Control and Salt stress conditions, and between ER and ERHG, as determined by a two‐way repeated measures ANOVA (*p < 0.05, **p < 0.01). FW, fresh weight. ER, ‘Esprosso’; ERHG, ‘Esprosso High GABA’; FW, fresh weight; GABA, γ‐aminobutyric acid; SE, standard error.

Under salt stress, GABA concentration in ER fruits increased by 74.6%, from 0.92 to 1.60 mg g^−1^ FW, whereas ERHG fruits showed a 71.8% increase, from 1.42 to 2.45 mg g^−1^ FW (Figure 3A). The net stress‐induced increase was greater in ERHG than in ER. Two‐way ANOVA showed significant main effects of cultivar and stress, whereas their interaction was not significant (Table S1). These results indicate that ERHG maintained a higher GABA accumulation capacity under both control and salt stress conditions.

To examine whether the salinity‐induced increase in GABA concentration was associated with enhanced accumulation or a passive dehydration effect, GABA content per individual fruit was evaluated (Figure 3B). Under salt stress, GABA content per fruit in ER increased only slightly, from 18.12 to 18.34 mg, whereas ERHG showed a larger numerical increase, from 24.50 to 30.08 mg. Two‐way ANOVA showed a significant main effect of cultivar, but no significant effects of stress or interaction (Table S1). These results suggest that the increase in GABA concentration in ER was largely associated with a concentration effect, whereas ERHG showed a greater tendency to increase whole‐fruit GABA accumulation under salt stress.

### 3.4 Total Soluble Solids and Metabolic Trade‐Offs

**FIGURE 4:**
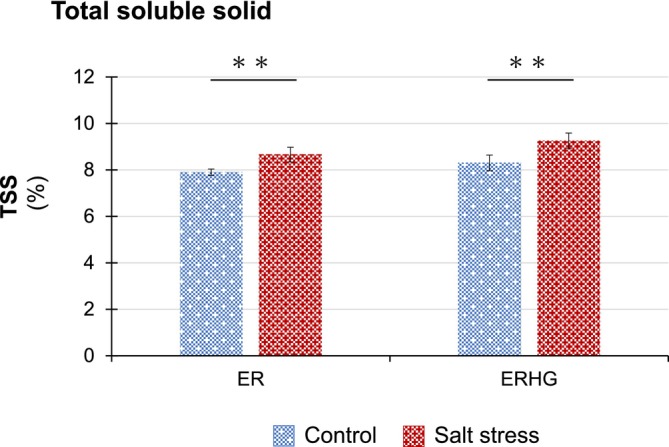
Effect of salt stress on total soluble solid on ER and ERHG. Red mature fruits were harvested from ER or ERHG plants grown under control or salt stress conditions and used for TSS measurement. Results are the mean ± SE (*n* = 5). Asterisks indicate significant differences between the control and salt stress conditions and between ER and ERHG, as determined by two‐way repeated measures analysis of variance (*p < 0.05, **p < 0.01). ER, Esprosso; ERHG, Esprosso High γ‐aminobutyric acid; TSS, total soluble solid; SE, standard error.

Enhanced GABA biosynthesis can theoretically reduce total soluble solids (TSS) due to carbon diversion from the TCA cycle. Conversely, salt stress is known to increase both GABA content and TSS. Given these opposing metabolic responses, salinity‐induced TSS attenuation was anticipated in high‐GABA cultivars. However, this trade‐off was not observed in a previous study using cherry tomato (Suzuki et al. 2025). Consistently, the present study using medium‐sized tomato lines demonstrated that salt stress significantly enhanced fruit TSS in both genotypes (Figure 4). While the cultivar main effect did not reach statistical significance, the associated effect size suggested a possible contribution of the high‐GABA trait to TSS under salt stress (Table S1). These results indicate that GABA accumulation mediated by *SlGAD3* modification does not negatively affect fruit TSS.

### 3.5 Proline and Ascorbic Acid Accumulation

**FIGURE 5:**
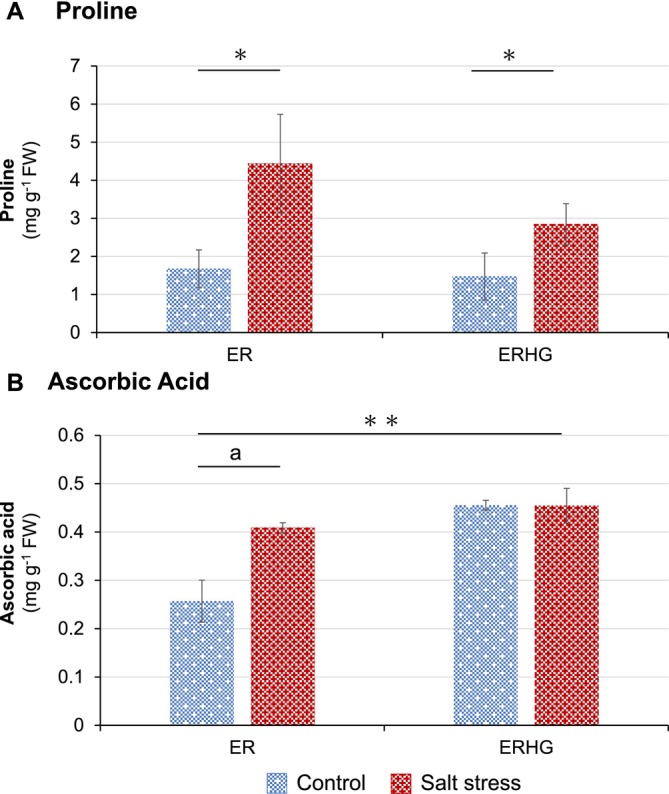
Proline and ascorbic acid contents in red‐ripe fruits of ER and ERHG plants grown under control and salt stress conditions. (A) Proline content (mg per gFW). (B) Ascorbic acid content (mg per gFW). Red mature fruits were harvested from ER or ERHG plants grown under control or salt stress conditions and used for proline and ascorbic acid measurement. Results are the mean ± SE (*n* = 5). Asterisks indicate significant differences between the control and salt stress conditions and between ER and ERHG, as determined by two‐way repeated measures analysis of variance (*p < 0.05, **p < 0.01). ER, Esprosso; ERHG, Esprosso High γ‐aminobutyric acid; SE, standard error.

To assess whether enhanced GABA biosynthesis interacts with osmotic adjustment and antioxidant‐related metabolism, proline and ascorbic acid concentrations were evaluated (Figure 5).

Under salt stress, fruit proline concentration increased by 165.3% in ER and by 93.5% in ERHG (Figure 5A). Two‐way ANOVA showed a significant main effect of stress, whereas the main effect of genotype and their interaction were not significant (Table S1). These results demonstrate that salt stress consistently stimulated proline accumulation across both genotypes, although the relative increase was smaller in ERHG than in ER.

Ascorbic acid content exhibited a genotype‐dependent response to salt stress (Figure 5B). Under control conditions, ERHG fruits maintained a higher baseline ascorbic acid content than ER fruits. Salinity induced a 59.0% relative increase in ER fruits, whereas ascorbic acid content remained virtually unchanged in ERHG fruits. Two‐way ANOVA showed a significant main effect of salt stress and a significant genotype × stress interaction, while the genotype main effect was not significant (Table S1). Tukey's HSD test showed that control ER fruits had significantly lower ascorbic acid levels than the other treatment groups, with no significant differences detected among control ERHG, stressed ER, and stressed ERHG fruits.

## 4 Discussion

Salt stress generally suppresses plant growth and productivity by inducing osmotic stress, ion imbalance, and oxidative stress (Munns and Tester 2008). However, the effects of salt stress depend on the severity and duration of stress, as well as plant developmental stage. In tomato, salt stress responses vary among cultivars and are associated with changes in antioxidant capacity and metabolic adjustment (Zushi and Matsuzoe 2009). In the present study, salt treatment increased fruit number in both ER and ERHG, and this increase was more pronounced in ERHG than ER (Figure 1B). These results suggest that the applied salt condition did not simply suppress reproductive development but may have induced an adaptive response. The enhanced fruit production observed in ERHG indicates the elevated GABA accumulation may contribute to the regulation of fruit development under salt stress.

GABA is recognized not only as a stress‐responsive metabolite but also as an important regulator of primary metabolism (Bouché and Fromm 2004; Bown and Shelp 2016). In plants, GABA is synthesized from glutamate through glutamate decarboxylase (GAD) and is further metabolized through the GABA shunt, which connects nitrogen metabolism with TCA cycle. Therefore, modification of GABA metabolism may influence carbon and nitrogen allocation, energy metabolism, and stress adaptation. In the present study, ERHG accumulated higher levels of GABA (Figure 3B) under salt stress and showed greater increases in fruit dry matter content compared with ER (Figure 2B). Notably, TSS showed a similar increase in both genotypes, suggesting that the increased dry matter accumulation in ERHG was not solely attributed to soluble sugars. Instead, enhanced GABA metabolism may have affected the accumulation of other metabolites or structural components, leading to increased fruit biomass.

The relationship between GABA accumulation and fruit quality‐related metabolism was further supported by changes in antioxidant‐related metabolites. ERHG maintained higher ascorbic acid levels than ER under both control and salt stress conditions (Figure 5B). Ascorbic acid plays a central role in maintaining cellular redox homeostasis by scavenging ROS (Foyer and Noctor 2011). Therefore, the higher ascorbic acid content in ERHG may indicate enhanced antioxidant capacity or improved maintenance of cellular homeostasis.

In contrast, the increase in proline content under salt stress was smaller in ERHG than in ER (Figure 5A). Proline is an osmoprotectant synthesized from glutamate and represents a key adaptive response to salinity (Szabados and Savouré 2010). Glutamate also serves as the direct precursor for GABA synthesis (Liu et al. 2011). Therefore, the enhanced flux toward GABA synthesis via elevated GAD3 activity in ERHG may have resulted in competitive partitioning of the shared glutamate pool between proline and GABA biosynthesis (Figure 5A). These findings are consistent with previous studies reporting that exogenous GABA enhances antioxidant enzyme activities and alleviates salt stress‐induced oxidative damage (Wu et al. 2020; Ullah et al. 2023).

Collectively, these findings suggest that fruit‐specific enhancement of GABA accumulation influences multiple aspects of tomato fruit metabolism under salt stress. Rather than directly conferring stress tolerance, elevated GABA levels may contribute to improved metabolic adjustment, allowing plants to maintain fruit development and quality under unfavorable conditions. However, the mechanisms underlying the association between GABA accumulation and improved metabolic adjustment remain to be elucidated. Further analyses of ROS accumulation, antioxidant enzymatic activities, and GABA‐shunt‐related metabolic changes will be necessary to clarify whether enhanced GABA accumulation directly contributes to stress adaptation or indirectly affects stress‐responsive pathways. From an applied perspective, metabolic modification of fruit GABA content using genome editing approaches may provide a promising strategy for developing crops with improved nutritional value and stable productivity under environmental stress.

## Funding

This work was supported by the Japan Science and Technology Agency (JST) ASPIRE program (JPMPAP24A3) to H.E.; The United States National Science Foundation (NSF) (OISE‐2434687) to H.E.; and the Special Joint Research Project between the University of Tsukuba and Sanatech Life Science Co. Ltd. (SDE01002) to H.E.

## Conflicts of Interest

T.S. declares no competing financial interest. M.T. and H.E. are employees of Sanatech Life Science Co. Ltd., which also provided partial funding for this study. However, all authors declare no conflicts of interest that could have influenced the findings or interpretation of the reported experiments.

## Supporting information


**Table S1:** Summary of two‐way ANOVA statistics (*F* values, *p* values) and effect sizes (*ηp* ^2^, Cohen's *d*) for yield, fruit number, fruit fresh weight, dry matter content, GABA concentration, GABA content per fruit, TSS, proline, and ascorbic acid across cultivar and salt stress treatments.

## Data Availability

The data that supports the findings of this study are available in the Supporting Information of this article.
